# Global scale assessment of urban precipitation anomalies

**DOI:** 10.1073/pnas.2311496121

**Published:** 2024-09-09

**Authors:** Xinxin Sui, Zong-Liang Yang, Marshall Shepherd, Dev Niyogi

**Affiliations:** ^a^Maseeh Department of Civil Architectural and Environmental Engineering, Cockrell School of Engineering, The University of Texas at Austin, Austin, TX 78712; ^b^Department of Earth and Planetary Sciences, Jackson School of Geosciences, The University of Texas at Austin, Austin, TX 78712; ^c^Department of Geography, University of Georgia, Athens, GA 30602

**Keywords:** urban sustainability, global change, rainfall extremes, cities

## Abstract

Urbanization has accelerated dramatically across the world over the past decades. Urban influence on surface temperatures is now being considered as a correction term in climatological datasets. Although prior research has investigated urban influences on precipitation for specific cities or selected thunderstorm cases, a comprehensive examination of urban precipitation anomalies on a global scale remains limited. This research is a global analysis of urban precipitation anomalies for over one thousand cities worldwide. We find that more than 60% of the global cities and their downwind regions are receiving more precipitation than the surrounding rural areas. Moreover, the magnitude of these urban wet islands has nearly doubled in the past 20 y. Urban precipitation anomalies exhibit variations across different continents and climates, with cities in Africa, for example, exhibiting the largest urban annual and extreme precipitation anomalies. Cities are more prone to substantial urban precipitation anomalies under warm and humid climates compared to cold and dry climates. Cities with larger populations, pronounced urban heat island effects, and higher aerosol loads also show noticeable precipitation enhancements. This research maps global urban rainfall hotspots, establishing a foundation for the consideration of urban rainfall corrections in climatology datasets. This advancement holds promise for projecting extreme precipitation and fostering the development of more resilient cities in the future.

## Significance

This research reports a global analysis of urban precipitation anomalies encompassing over one thousand cities worldwide. While earlier studies have focused on the impact of urbanization on precipitation for specific cities or isolated thunderstorm cases, our research breaks innovative ground by mapping global urban precipitation hotspots over the past 20 y. This study provides global evidence of noticeable urban precipitation anomalies, especially in hot and humid climates. Beyond the anticipated influence of local climate, our findings reveal that higher levels of urbanization enhance these urban precipitation anomalies. This research not only deepens our understanding of how cities shape precipitation but also establishes the groundwork for incorporating urbanization considerations into future precipitation projections.

Urbanization continues to be a dominant global driver of land cover and land-use change. In urban areas, the rapidly growing population faces heightened exposure and vulnerability to natural hazards, including but not limited to extreme heat and floods (1). This susceptibility is exacerbated by the rapid pace of urbanization and concurrent anthropogenic activities, such as the emission of greenhouse gases and air pollutants. Scientific evidence underscores the impact of these urbanization-related factors on long-term climate patterns and the occurrence of extreme weather events (2, 3).

As one of the most complex land cover changes, urbanization is evidenced to affect the formation, movement, and enhancement of precipitation by modifying the regional atmospheric boundary layer (4, 5). Urban heat islands, a notable climate feature of urbanization, have been noted to contribute to precipitation dynamics. Notably in the summer season, the dynamical feedback of urban heat islands leads to enhanced wind updraft and surface convergence because of heat flux gradients in the boundary layer, fostering a conducive setting for convective rainfall, particularly on the leeward side of cities (6, 7). Considering the urban morphology, tall and dense buildings in urban areas increase surface roughness. This heightened roughness can either slow down air flow, prolong the duration of precipitation (8), or enhance surface convergence and alter the storm structure (9). Conversely, the replacement of substantial vegetative cover and permeable soil surfaces with impermeable structures diminishes evapotranspiration, reducing regional atmospheric moisture and impeding urban precipitation development (10). Anthropogenic aerosols also affect urban precipitation through microphysical processes. These aerosols serve as cloud condensation nuclei and ice nuclei, aiding precipitation initiation, yet they can also hinder precipitation by reducing moisture content and cooling the atmosphere, which increases atmospheric stability and impedes the precipitation process (11, 12).

Past studies have extensively explored the diverse impacts of urbanization on precipitation in various cities, including Atlanta, Beijing, Houston, Indianapolis, Mexico City, Phoenix, and San Miguel de Tucumán (10, 13–18). A meta-analysis of different studies has revealed some shared aspects of rainfall changes but also identified several unresolved issues (19). A crucial gap identified is the lack of a systematic study encompassing global cities across a wide range of geographic and climatological conditions. The current study addresses this need for a comprehensive examination and identifies the global urban precipitation hotspots through a comparative analysis of annual precipitation amount and daily extreme precipitation between global cities and their adjacent rural areas.

We use a global satellite-based precipitation dataset of high spatial resolution (0.1° × 0.1°), Integrated Multi-satellitE Retrievals for Global Precipitation Measurement (IMERG) (20), to examine daily precipitation anomalies for over one thousand cities worldwide from 2001 to 2020. Recognizing the uncertainty in the satellite precipitation product, a radar-based National Centers for Environmental Prediction (NCEP) Stage IV precipitation dataset (21) is also used to calculate urban precipitation anomalies for US cities. This additional dataset serves for comparison and quality assurance.

We identify the global urban and build-up land cover based on MODIS satellite observations. We exclude the cities with an urban radius smaller than 5 km, as smaller cities may not exert a significant impact on local rainfall (11), and the 0.1° resolution of precipitation data may not adequately capture features in smaller areas. For each city, recognizing that urban influences on precipitation extend beyond city boundaries, we establish three rural domains (R1, R2, and R3) at distances of one, two, and three times the urban radius from the city edge. We assume that the farthest rural domain (R3) lies out of the dominant range of city influence. We compare annual precipitation amounts, extreme precipitation, and changes over 20 y in urban and rural areas. By investigating global urban precipitation anomalies, this study provides insights into understanding anthropogenic effects on urban climate and projecting extreme precipitation in cities.

## Results

### Mean Annual Precipitation Anomalies.

**Fig. 1.**
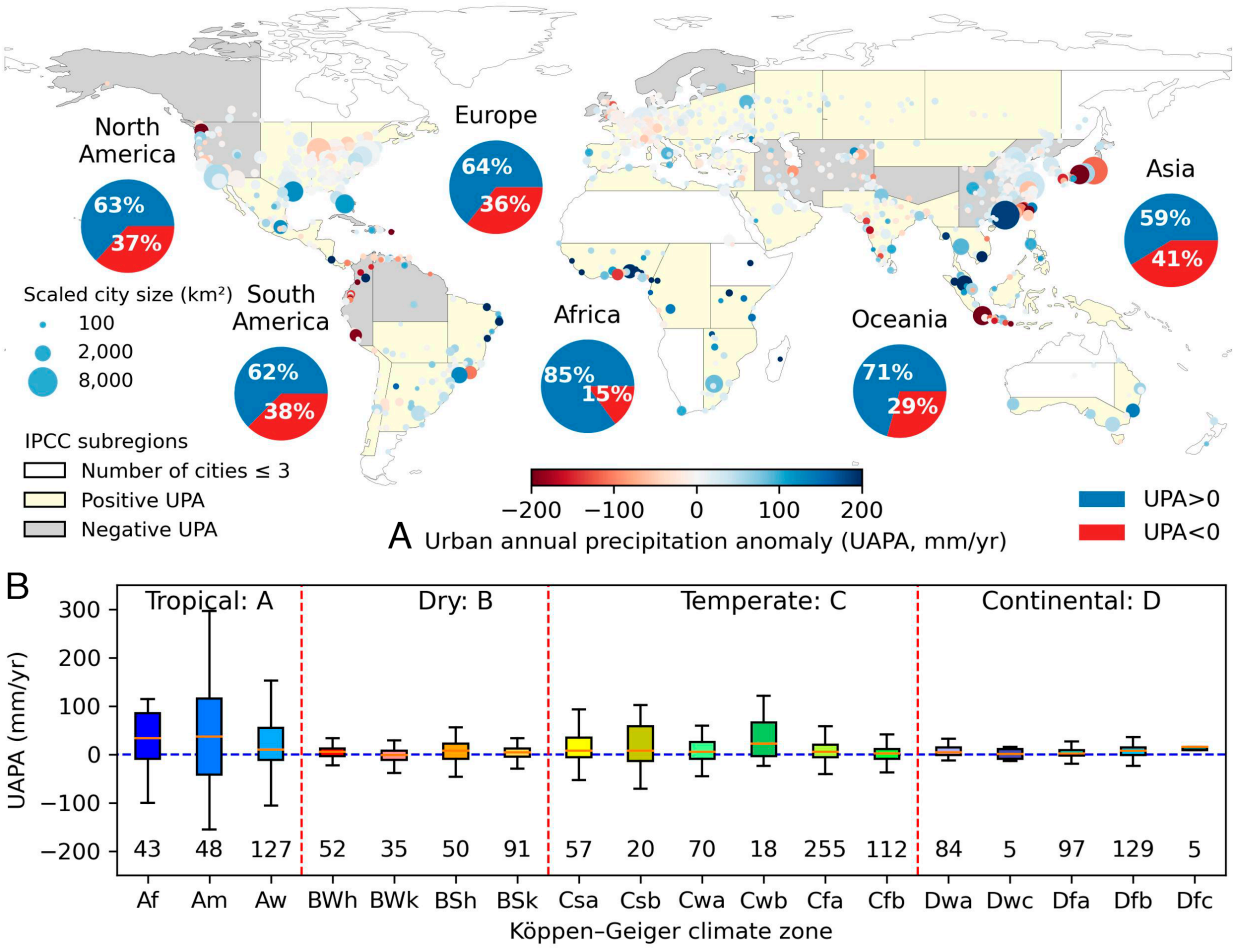
Global urban annual precipitation anomalies. The urban annual precipitation anomalies according to continents and climate zones. (*A*) The color of the base map shows that the average of urban precipitation anomalies in the IPCC subregion is positive or negative. Each dot on the base map indicates one city, and the size and color of the dot describe the size and precipitation anomaly extent of the city. The pie charts show the percentage of cities with positive and negative (more or less precipitation over urban grids) urban precipitation anomalies in each continent (the pie charts for each IPCC subregion are shown in *SI Appendix*, Fig. S15). (*B*) The box plot for urban annual precipitation anomalies for cities in different climate zones. The numbers above the axis are the number of cities in that climate zone.

We investigate 1,056 cities around the world and find that 63% show urban wet islands, which means more precipitation in cities than in the peripheral rural areas. Fig. 1*A* shows a spatial map of global urban annual precipitation anomalies. The urban precipitation anomalies differ among continents (Fig. 1*A*). The highest proportions of positive urban precipitation anomalies were noted for the cities in Africa and Oceania. Overall, 85% and 71% of African and Oceania cities have more mean annual precipitation than their rural controls, which could be influenced by urban development and local climate and topography conditions such as high temperatures and elevation difference. To accurately describe the magnitude of urban precipitation anomalies, we consider both the actual precipitation differences and the percentage differences. This is because, for some dry cities with small precipitation values, percentage differences can come out to be very large values, which is misleading for the context of analysis. On the other hand, for the wet cities, percentage differences are deemed more appropriate for comparing and communicating the findings.

We find that 17 cities show significant urban wet islands with urban precipitation anomalies above 200 mm per year (8.3 to 85.3%), and of these, nine are located in Africa (*SI Appendix*, Fig. S1). In contrast, we find a relatively low fraction of urban wet islands in Asia, nonetheless with a robust 59% of cities having positive urban precipitation anomalies. Interestingly, 12 cities exhibit apparent urban dry islands with urban precipitation anomalies smaller than −200 mm/y (−15.8 to −9.7%), eight of which are in Asia. Further, we noted a spatial pattern in urban precipitation anomalies across North and South America: Most of the cities in western mountainous areas are drier than their nearby rural areas with less annual precipitation, while cities on the east plains are wetter than nearby rural areas. This highlights that in the western mountainous areas, precipitation patterns are influenced by orographic conditions. With cities often situated in valleys and lowlands, the nearby rural hillsides receive more orographic precipitation, resulting in noticeable negative urban precipitation anomalies. It is worth mentioning that previous studies have indicated a persistent downwind urban precipitation enhancement for mountainous and complex topography (14, 17, 22). Our findings of negative urban precipitation anomalies based on the urban–rural comparison may therefore be influenced by elevation differences, rather than implying the absence of urban precipitation enhancement. This underscores the importance of considering background climates and geographical conditions in addressing the urban precipitation problem.

Fig. 1B shows urban annual precipitation anomalies according to different climate zones. We find that the tropical and temperate climate zones with larger annual precipitation amounts have larger urban annual precipitation anomalies than the dry and continental climates (*SI Appendix*, Fig. S2*B*). More than 70% of the cities in the tropical rainforest (Af) and warm-summer humid continental climate (Dfb) show urban wet islands, and these two climate zones have a commonality of no dry season. Different from other climate zones, the majority of cities in the cold desert climate (Bwk) have less precipitation than nearby areas, which are typically at high altitude mountainous areas. To quantify the relationship between urban precipitation anomalies and background wetness and temperature, we conduct a multivariable linear regression to predict the average positive and negative urban precipitation anomalies based on the average temperature and precipitation in the surrounding rural areas for cities in different climate zones (*SI Appendix*, Fig. S2*A*). Both the positive and negative regressions exhibit satisfactory fits (R^2^ of 0.54 for urban wet islands, 0.74 for urban dry islands) and reveal larger precipitation anomalies between urban and rural scenarios under wetter and hotter environments.

Further, we consider that the urban precipitation anomalies, much like urban heat islands (23, 24), can be linked to urban development (11). A key indicator of urban development is the size of the urban footprint. We find that 67% of the larger half of cities exhibit urban wet islands compared to 58% in the smaller half. When cities showing urban wet and dry islands are separated, we observe a yearly increase of 1.4 mm in the magnitude of urban wet islands (*SI Appendix*, Fig. S3). That is the average annual precipitation anomaly almost doubled from 37 mm in 2001 to 62 mm in 2020 for each city, while the average magnitude of urban dry islands remains consistent at around 37 mm per year. This increase in positive urban precipitation anomalies is attributed to both urban development and changes in regional meteorological conditions within the past two decades. Additionally, we note a sublinear increase in average urban precipitation anomalies with the increase in the population (*SI Appendix*, Fig. S3).

Additionally, the presence of background complex topography such as mountainous or coastal factors also contributes to a large magnitude of urban precipitation anomalies. Of the 17 cities with significant urban wet islands (UPA > 200 mm/y), 10 of them are coastal cities (*SI Appendix*, Figs. S1, S4). The only large city (city area > 95th percentile) is Kuala Lumpur, Malaysia, where the average annual precipitation anomaly is 247 mm higher (10.3%) relative to its nearby rural areas. As for negative urban precipitation anomalies, we also find notable urban dry islands (UPA < −200 mm/y) over 12 cities, of which 11 were in coastal regions (*SI Appendix*, Fig. S4). Four of them are large cities in complex terrains: Seattle, in the United States (−14.8%), Quanzhou in China (−14.3%), Himeji-Osaka-Kyoto in Japan (−11.0%), and Jakarta in Indonesia (−7.5%).

### Extreme Precipitation Anomalies.

**Fig. 2.**
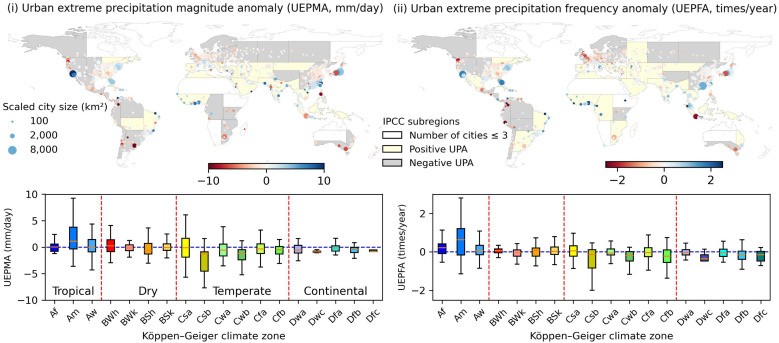
Global urban extreme precipitation anomalies. This figure is similar to Fig. 1 but for the urban extreme precipitation anomaly. The urban extreme precipitation magnitude (*i*) and frequency (*ii*) anomalies for global cities according to different climate zones. According to Köppen climate classification, the third letter, “a,” “b,” and “c,” indicates hot, warm, and cold summer, and “h” and “k” indicate hot and cold. Relative to the cities in cold climates or have cold summers, the cities in hot climates or have hot summers have larger urban extreme precipitation anomalies (e.g., Csa has higher urban extreme precipitation anomalies compared to Csb in Fig. 2, *i*).

We define extreme precipitation as the value equal to or exceeding the 95th percentile of maximum daily precipitation in this research. The extreme precipitation magnitude and frequency anomalies are investigated for global cities (Fig. 2). The satellite-based IMERG precipitation dataset indicates that 56% (51%) of global coastal cities and 40% (32%) of global inland cities are experiencing more frequent (larger magnitude) extreme precipitation compared to nearby rural areas. Given that the estimation of extreme precipitation from IMERG satellite datasets is often challenging (25), a comparison is done for the US cities using a radar-based Stage IV precipitation dataset. The Stage IV radar data are available over the continental US as a 4 km grid product and can be considered to be more representative of local changes in rainfall compared to IMERG. The radar dataset shows that 65% (58%) and 51% (53%) of US coastal and inland cities are experiencing more frequent (larger magnitude) extreme precipitation (*SI Appendix*, Fig. S5). The two precipitation datasets show different extents of urban extreme precipitation anomalies, but both results similarly show that coastal cities are more likely to experience positive urban extreme precipitation anomalies compared to inland cities.

As noted, both Oceanian and African cities show more annual precipitation than peripheral rural areas but yield different urban extreme precipitation anomalies. Oceania cities tend to have negative urban extreme precipitation anomalies, which means that they do not have more frequent nor larger magnitude extreme precipitation compared to nearby rural areas. African cities show notable extreme precipitation anomalies for both high frequency and large magnitude, especially the West African coastal cities such as Lagos and Accra. Most of the north and middle European cities do not experience more frequent and larger magnitude extreme precipitation relative to rural areas. Yet, some large cities in southern Europe such as Milan, Naples, and Barcelona show notable positive urban extreme precipitation anomalies. Urban extreme precipitation anomalies in North and South America show a similar spatial pattern as the annual precipitation anomalies, that is negative in the western dry mountains and positive in the eastern wet and relatively flat regions. Compared to the inland cities, the chance of positive urban extreme precipitation anomalies increases remarkably for coastal cities in North America and Asia.

Fig. 2 compares urban extreme precipitation anomalies according to different climate zones. Similar to annual precipitation anomalies, tropical cities have more significant extreme precipitation anomalies compared to other climate zones. Over 70% of the cities under the tropical monsoon climate (Am) have more frequent and higher magnitude extreme precipitation compared to the nearby rural areas. Second, among each main climate group, the cities experiencing hot conditions such as hot summers are more likely to have large urban extreme precipitation magnitude anomalies than the cities under cold conditions or have cold or warm summers (e.g., Csa and Csb in Fig. 2, *i*). This indicates that high temperatures can be positively linked to extreme precipitation events. Correspondingly, the cities with cold or warm summers or in the cold desert have less frequent and smaller magnitudes of extreme precipitations (e.g., Dwa and Dwb in Fig. 2, *ii*).

### City Effects on Surrounding Precipitation.

**Fig. 3.**
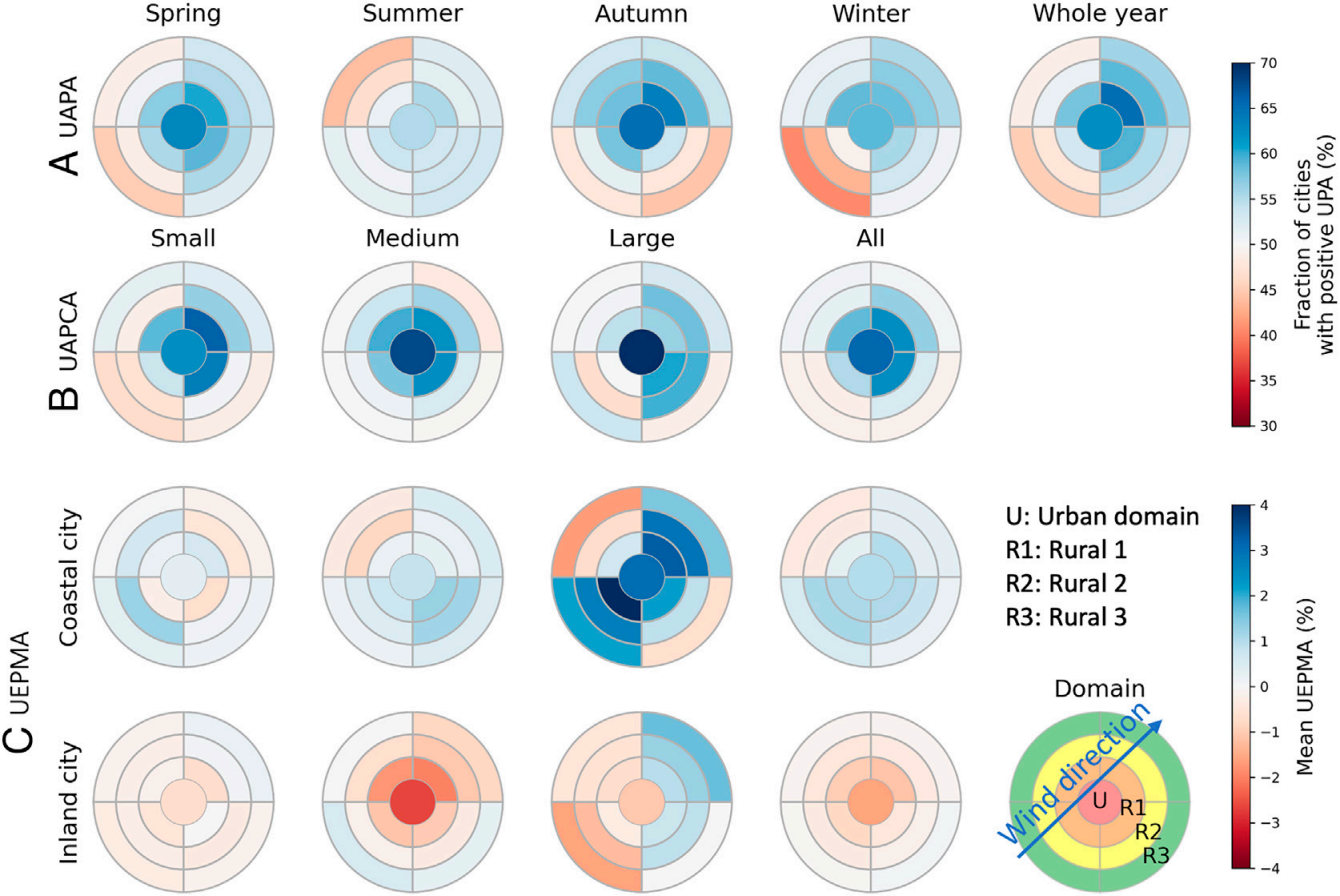
Wind direction influences urban precipitation anomalies. The spatial pattern of urban precipitation anomalies in urban (U) and three rural domains (R1, R2, and R3) relative to wind direction. (*A*) The seasonal variation of urban annual precipitation anomalies (UAPA) in urban and three rural domains. (*B*) Urban annual precipitation change anomalies (UAPCA) according to different city sizes. Small, medium, and large cities are defined as city areas smaller than 50th percentile, between 50th and 90th percentile, and larger than 90th percentile. (*C*) Urban extreme precipitation magnitude anomalies (UEPMA) for coastal and inland cities according to different city sizes.

Fig. 3 further reveals the precipitation anomalies in urban areas and peripheral rural areas. It is well known that the dynamic effect of the urban area extends the influence on precipitation also to the peripheral rural areas (18). As seen in Fig. 3*A*, the urban domain shows a positive annual precipitation anomaly, as well as its peripheral rural areas within a distance equivalent to the urban radius (R1). In particular, when considering the wind direction, the downwind side has an even more obvious precipitation hotspot than the urban domain (*SI Appendix*, Fig. S6). The downwind rural areas (R2 and R3) continue to have positive annual urban precipitation anomalies, while the upwind side has negative anomalies.

The seasonal variation in the urban precipitation anomalies is also examined. Over 63% and 65% of global cities have positive urban precipitation anomalies in spring and autumn, whereas 55% and 59% are in summer and winter. Focusing on the positive urban precipitation anomalies, we find that summer and autumn show larger urban precipitation enhancement. On average, each city receives 12.8 mm (6.9%) and 11.3 mm (4.4%) more precipitation per summer or autumn than rural control areas (R3), while this number is below 9.7 mm (22.9%) and 8.3 mm (4.7%) for winter and spring. One explanation is that during hot seasons, the heat supports the development of local convective precipitation around urban low-pressure centers (5).

While we are constrained by the 20-y data period, it is known that rapid urbanization has occurred in more recent years. Therefore, we undertake a simple comparison of the precipitation anomalies for the first versus second 10-y period. We find that the changes in urban and rural precipitation show a similar large-scale spatial pattern, such as a decrease in South America and central Europe and an increase in the eastern US (*SI Appendix*, Fig. S7). Calculating the difference between urban and rural precipitation change (*SI Appendix*, Fig. S8), we study the urban precipitation change anomaly according to the size of city footprints. We find that urban domains and their peripheral rural areas (R1) not only show a tendency to receive more rain but are also becoming wetter at a rate higher than surrounding rural control areas (R3), especially for cities with large footprints (Fig. 3*B*). This result has obvious implications to the increased risk in urban flooding witnessed in many cities across the globe.

The coastal and Inland cities show different results for urban extreme precipitation anomalies. The coastal cities could experience a larger magnitude of extreme precipitation compared to the nearby rural control areas (R3), while the inland cities have a smaller magnitude of extreme precipitation on average (Fig. 3*C*). However, the downwind side of both the large coastal and inland cities (city footprint ≥ 90th percentile) experiences positive extreme precipitation magnitude anomalies. The extreme precipitation of 95th percentile in the nearest downwind rural domain is 2.3% larger than it is in the rural control areas for large coastal cities, while it is 0.8% for large inland cities. In summer, both the large inland and coastal cities (and their downwind rural areas) show a large positive extreme precipitation anomaly, which is 3.5% for inland cities and 3.0% for coastal cities.

### The Influences of Other Environmental and Urbanization Conditions.

We investigate the relationship between several environmental and urbanization factors (topography, temperature, wetness, population, built-up area, aerosols, and urban heat islands) and urban precipitation anomalies using Spearman’s correlation coefficients. By separating the cities showing urban wet and dry islands, we find that the urban elevation variability (or topographic relief) is an important factor in the negative precipitation anomalies (*SI Appendix*, Fig. S9). That is, the cities in the lowland or valley receive less annual precipitation than the surrounding rural areas, while the cities on flat plains or highlands are more likely to experience positive annual precipitation anomalies. To further investigate the influence of topography on precipitation, we group cities based on their elevation relief. We noted that cities with higher elevation relief exhibit larger urban precipitation anomalies, while valley cities with elevation relief below −200 m have an average of −18.5 mm less annual precipitation compared to their rural backgrounds (*SI Appendix*, Fig. S10). This result confirms our earlier assertion of negative precipitation anomalies in western mountainous cities and positive precipitation anomalies in eastern regions across North and South America.

Other notable environmental factors include local temperature and wetness. The annual mean temperature and precipitation in the surrounding rural areas are calculated to reflect the background temperature and wetness conditions. We find that the temperature and wetness conditions are positively related to the positive urban precipitation anomalies and negatively related to the negative urban precipitation anomalies (*SI Appendix*, Fig. S9). This aligns with the outcomes of the multivariable linear regression for cities in different climate zones as cities in hot or wet climates tend to have larger precipitation anomalies compared to cities in cold or dry regions.

**Fig. 4.**
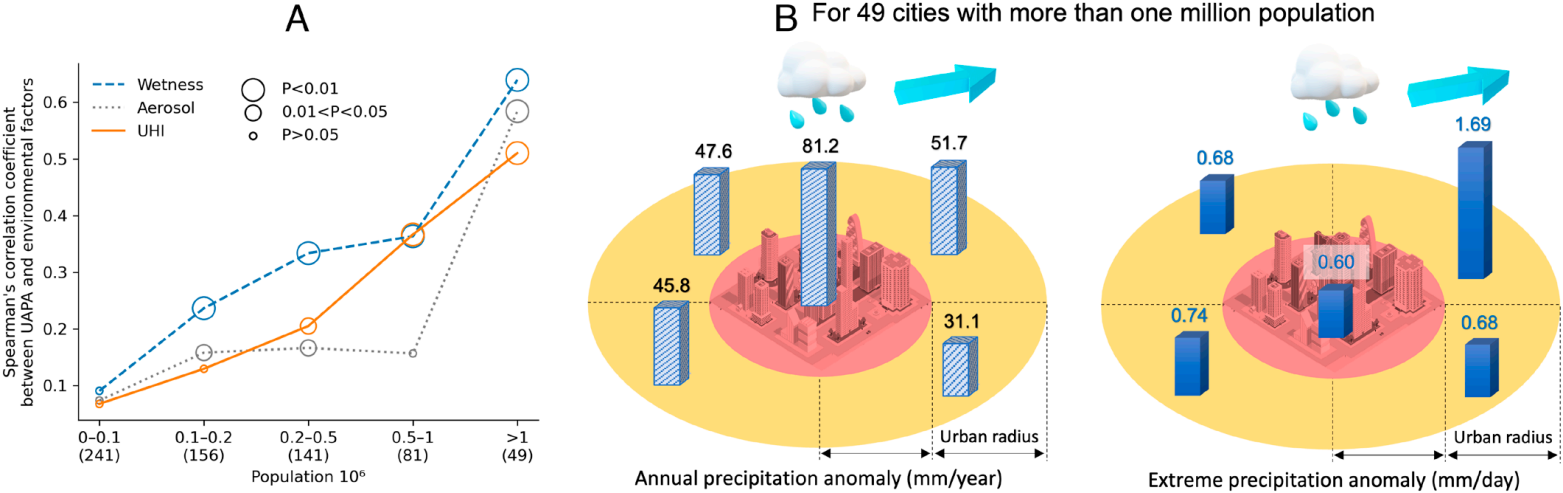
The influence of environmental and urbanization factors. (*A*) Correlation between urban annual precipitation anomalies (UAPA) and different environmental factors, including urban heat islands (UHI), aerosol, and local wetness, for the cities showing urban precipitation enhancements. The cities are identified in five groups per population size, and the numbers in the parenthesis along the x-axis indicate the number of cities in each group. The linear regression between the UAPA and UHI for each population group is shown in *SI Appendix*, Fig. S12. (*B*) The average annual and extreme precipitation anomaly in the cities and the surrounding rural areas for the 49 cities with more than one million population. Results show that the urban annual precipitation hotspot is within cities, while the urban extreme precipitation hotspot is in the downwind urban areas.

The population has the largest correlation with the urban annual precipitation anomaly compared to other environmental and urbanization factors, whose correlation coefficient is 0.46 for 668 global cities showing urban precipitation enhancement (*SI Appendix*, Fig. S9*B*). In addition to dense population, urban areas are also major sources of aerosol and anthropogenic heat. To quantify these aerosol and temperature conditions, we calculate the average values of aerosol optical thickness around each city and daytime surface urban heat islands over 20 research years (*SI Appendix*, Fig. S11). We study the influences of urban aerosol and urban heat islands on precipitation by clustering the 668 cities into five groups according to the population (Fig. 4*A*). Results indicate that aerosol optical depth and surface urban heat islands have large correlation coefficients with the annual precipitation enhancement, especially for the cities with high populations. The aerosols can act as cloud condensation nuclei (26), and urban surface heating aids mesoscale convergence and convective precipitation (13, 27).

For the 271 cities with population more than 20,000, whose urban precipitation anomalies are significantly related to the urban heat islands in Fig. 4*A*, we find that for each one-degree increase in surface urban heat islands, there is 24.7 mm more annual precipitation in the urban precipitation anomalies. The larger the population, the larger the urban precipitation anomalies as well as the urban heat island (*SI Appendix*, Fig. S12). We also calculate the average annual and extreme precipitation anomaly for these 49 megacities with population more than one million under the high effects of urban aerosol and anthropogenic heat (Fig. 4*B*). Here, we find that the urban annual precipitation hotspot is within cities, while the urban extreme precipitation hotspot is located in the downwind urban areas. The average annual precipitation enhancement within these megacities is 81 mm per year (7.2%), which is 42 mm per year (3.6%) for the peripheral rural area (R1). As for the extreme precipitation, the downwind rural areas have a larger magnitude of extreme precipitation than the cities. The average extreme precipitation as the 95th percentile of daily precipitation in the downwind peripheral rural areas is 1.69 mm/d (3.8%) larger than the rural control areas, which is 0.6 mm/d (1.2%) larger for urban areas.

Previous research has documented the role of wind in urban precipitation anomalies and reported positive anomalies both over cities and downwind sides. The variation of rainfall hotspots could result from different combinations of urban heat islands and wind speeds, because low wind speeds and strong urban heat islands may cause convergence within cities, while strong wind and low urban heat islands help spread rainfall downwind (28). Here, the different hotspot locations concerning precipitation amount and extreme precipitation underscore the importance of considering the mechanisms of precipitation. Certain storms are more prone to causing extreme wet conditions, and urbanization may impact various storms differently. Further studies could explore the interaction between urban heat islands and wind impacts of rainfall under different precipitation mechanisms.

## Discussion

This research employs a high-resolution (0.1°) IMERG precipitation dataset derived from satellite product (20) to examine global urban precipitation anomalies. The satellite-based precipitation product benefits from its extensive observation coverage. In the literature, the selection of the IMERG precipitation product for global analysis has been emphasized over other satellite and reanalysis datasets, with no systematic errors aligned to urban areas reported for IMERG based on extensive evaluations (29). However, it is essential to acknowledge a known limitation of IMERG and other global products, which is the underestimation of extreme precipitation (25). This limitation poses a challenge when evaluating urban extreme precipitation anomalies in this research. To understand the uncertainty and develop a robust analysis of urban precipitation anomalies, we calculate urban precipitation anomalies for US cities using more accurate radar-based Stage IV datasets (21). Compared with the Stage IV results, we find that the IMERG dataset can capture an accurate pattern of urban annual precipitation anomalies with satisfactory F1 scores (*SI Appendix*, Fig. S5), which analysis constitutes the primary findings of this research. While more differences are noted in IMERG when calculating urban extreme precipitation anomalies compared to Stage IV, we find a relatively high accuracy for cities with large urban footprints. Consequently, we categorized cities based on different urban footprints when presenting extreme precipitation anomalies, focusing particularly on large cities. Moreover, we emphasize robust indicators such as city percentages and qualitative intercomparisons (inland or coastal cities or cities in different climate zones) rather than relying solely on absolute values in our results.

Other limitations in the analysis stem from the determination of urban and rural domains. This research uses the land cover type data for 2020 to determine the urban and rural domains. We focus on the spatial comparison of precipitation in urban and rural areas rather than the land cover change during the two decades of research years. For each city, we consider three rural domains (R1, R2, and R3) of different distances from the urban edges, where R3 is assumed to be out of the radius of urban effects as the rural control areas. This definition of rural control follows prior work on this topic which analyzes the storm dynamics for specific storm cases. Since cities in different regions may experience different rates of urbanization for the 20-y period being researched, the study could overestimate the urbanization condition for those cities that developed rapidly during the past two decades. Consequently, the urban impacts on precipitation could be underestimated when trying to quantitatively address the urban-related precipitation changes.

We study global urban precipitation anomalies and conclude that the urban and its peripheral rural areas, particularly the downwind side, receive more precipitation than their surrounding rural background. Larger cities show more apparent urban wet islands. In addition, we find several climatological and topographic characteristics related to urban precipitation anomalies. First, positive urban precipitation anomalies are collocated to high-temperature regions since large urban precipitation and extreme precipitation anomalies are found in tropical zones or regions with hot summers. In addition, coastal cities are likely to experience larger urban extreme precipitation anomalies compared to inland cities, since the moisture source from coastal areas is found to potentially enhance urban precipitation anomalies. Finally, urban precipitation anomalies show differences according to different local topography with negative anomalies for mountainous cities versus positive anomalies for cities in relatively flat terrain. These contrasting urban precipitation patterns are likely attributed to the elevation relief of cities and influenced by topography as the first-order effect ahead of urban feedback. This outcome underscores the need for further investigation into urban–sea and urban–mountain interactions, distinguishing urban influences from different climate and topographic conditions.

## Materials and Methods

### Urban and Rural Domains.

This research investigates urban precipitation anomalies for 1,056 global cities (*SI Appendix*, Fig. S13). As the urban impacts on precipitation are not constrained within urban regions, we analyze the precipitation for each city and its surrounding rural domains. Three rural concentric domains of different distances from the urban edges are determined. We assume that the precipitation in the rural domain 3 (R3) could be separate from urban influences and therefore use the precipitation in R3 as the rural control to reflect the normal precipitation without urban influences, following prior studies (9).

For each city, one urban and three corresponding rural domains are determined by the following procedures (*SI Appendix*, Fig. S14). First, the global urban built-up pixels are extracted from a 0.05-degree gridded MODIS land cover type data (MCD12C1). The urban pixels with at least one edge-adjacent are clustered as one individual city. Then, we calculate the urban radii for these clustered cities, assuming city shapes as circles. Building on the findings of city size impact rain (11), we consider that small cities may not generate sufficient impacts on daily precipitation nor be detected by the satellite precipitation data of 0.1-degree (11 km). Therefore, we remove the small cities with radii smaller than 5 km, and the remaining cities are designated as urban domains. For the remaining over 1,000 cities, three rural domains (R1, R2, and R3) are drawn as three concentric rings beyond the urban domain, whose distances from the urban domain edges are, respectively, between 0 to 1, 1 to 2, and 2 to 3 times urban radii. Since some cities are close to each other, for example, the urban domain of city A overlaps with the R2 and R3 domains of city B, in that case, the overlap region is considered as the urban domain of city A, rather than the R2 and R3 domains of city B. In this way, we designate the overlapped regions to the higher-level domain (closer to the urban). Finally, we rule out the water bodies or wetland surfaces from the urban and rural domains to avoid the influence of water surfaces.

### Urban Precipitation Anomaly Indicator.

The difference between precipitation in the urban and rural control areas is investigated using the precipitation anomaly indicators. We test several precipitation indicators to describe the general and extreme precipitation anomalies. For the general condition, the mean annual precipitation, daily rain ratio, daily precipitation intensity, and annual trend are tested. For the extreme precipitation, we first use the maximum 1 and 5-d accumulated precipitation, the annual longest consecutive wet and dry days, and 90th, 95th, 99th, and 99.9th percentile precipitation intensity. Finally, four urban precipitation anomaly (UPA) indicators are selected for the final investigation (Table 1), and the precipitation intensity of 95th percentile of wet days is used to define the extreme precipitation events.

1. The urban annual precipitation anomaly (UAPA) is the difference in mean annual precipitation between the urban domain and rural control areas for 20 y (2001 to 2020).
2. Two mean annual precipitation changes in the urban domain and rural control areas are calculated between 2001 to 2010 and 2011 to 2020, separately. Then, the difference between these annual precipitation changes is calculated as the urban annual precipitation change anomaly (UAPCA).
3. The extreme precipitation magnitudes are calculated as the daily precipitation of 95th percentile for urban and rural control areas, whose difference is determined as the urban extreme precipitation magnitude anomaly (UEPMA).
4. The daily precipitation of 95th percentile in the rural control areas is calculated as the threshold of extreme precipitation intensity for each city. Then, the precipitation intensities larger than this threshold are counted for urban and rural control areas, respectively. Since urban and rural areas are of different sizes, the area-normalized extreme precipitation frequencies are calculated for urban and rural control areas, whose difference is the urban extreme precipitation frequency anomaly (UEPFA).

**Table 1.** Four urban precipitation anomaly indicators used in the study

Type	UPA indicators	Full name	Unit
General precipitation condition	UAPA	Urban annual precipitation anomaly	mm/year
	UAPCA	Urban annual precipitation change anomaly	mm/year
Extreme precipitation	UEPMA	Urban extreme precipitation magnitude anomaly	mm/day
	UEPFA	Urban extreme precipitation frequency anomaly	occurrence/year

## Data Processing

This research uses the final run of the Global Precipitation Measurement (GPM) IMERG daily precipitation dataset (20) in 0.1° × 0.1° to analyze precipitation anomalies around global cities. The ground-based NCEP Stage IV precipitation product (21) is used for quality control, which provides precipitation data across the United States. Here, we aggregate the 6-h Stage IV precipitation dataset to a daily scale and perform averaged interpolation on the 4 km × 4 km gridded data to achieve a resolution of 0.1° × 0.1°.

In addition to precipitation data, we also use other environmental datasets from 2001 to 2020, including aerosol optical thickness, 850 hPa wind direction, and land surface temperature. The average aerosol optical thickness is calculated for global cities according to Level-3 Aura/OMI Global Aerosol Data (OMAEROe) (30). The wind direction at 850 hPa is retrieved from ECMWF Reanalysis v5 data (ERA5) (31). The climatologically dominant wind direction for each city is calculated by averaging the wind for four seasons. The calculated results are classified into four cardinal directions as northeast, northwest, southeast, and southwest to further determine the upwind and downwind for each city in each season. The daytime land surface temperature is retrieved from the Terra Moderate Resolution Imaging Spectroradiometer (MODIS) Land Surface Temperature/Emissivity Monthly (MOD11C3) (32). The mean annual daytime temperature in rural domain 1 (R1) is calculated to show the local temperature condition for each city. The magnitude of urban heat islands is calculated from the temperature difference between the mean daytime temperature in the urban domain and rural domain 1 (R1).

The land cover type and elevation information are retrieved from the Terra and Aqua combined MODIS Land Cover Climate Modeling Grid (MCD12C1) (33) and Global Multi-resolution Terrain Elevation Data 2010 (GMTED2010) (34). Based on the MODIS land cover type data in 2020, the urban and rural domains as well as the water surfaces are determined. In addition, we calculate urban elevation anomalies using the 30 arcseconds DEM data, which is the difference between average elevations in the urban domain and rural domain 3 (R3). The climate zone information is retrieved from the present (1980 to 2016) map of the Köppen–Geiger climate classification (35).

To reflect the urbanization condition of different cities, the population and built-up area are retrieved from the Global Human Settlement Layer (GHSL) (36), which provides global urbanization information for 1975, 1990, 2000, and 2014/2015. We accumulate the population and built-up area in the urban domain and rural domain 1 (R1) for each city in 2014/2015 to reflect the urbanization extent and type for different cities.

## Supplementary Material

Appendix 01 (PDF)

## Data Availability

Shapefile, NPY file data have been deposited in Zenodo (https://zenodo.org/record/8179621) (37).
