# Supplementary material for: Global scale assessment of urban precipitation anomalies
Source: Proc Natl Acad Sci U S A. 2024 Sep 9;121(38):e2311496121. doi: 10.1073/pnas.2311496121 (PMC11420207; doi:10.1073/pnas.2311496121)
Supplement: Supplementary file 1 — Appendix 01 (PDF) [file pnas.2311496121.sapp.pdf]

PNAS

Supporting Information for
Global Scale Assessment of Urban Precipitation Anomalies

Xinxin Sui, Zong-Liang Yang, Marshall Shepherd, Dev Niyogi

Dev Niyogi
Email: happy1@utexas.edu

This PDF file includes:

Figures S1 to S15
Tables S1 to S2

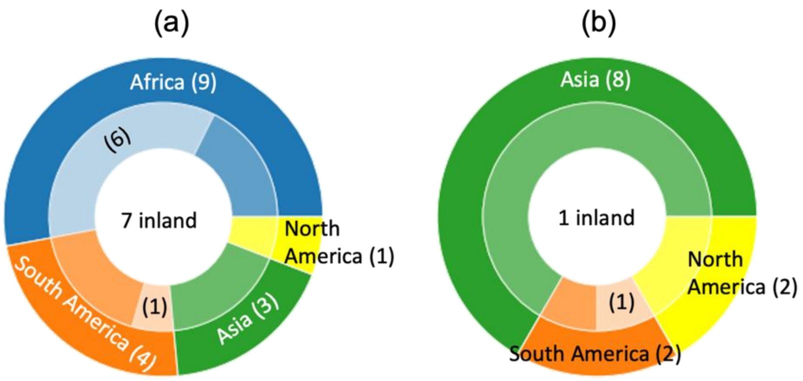

Fig. S1. The continental distribution of cities with large urban annual precipitation anomalies. (a) 17 cities with positive urban precipitation anomalies larger than 200 mm per year and (b) 12 cities with negative urban precipitation anomalies smaller than -200 mm per year.

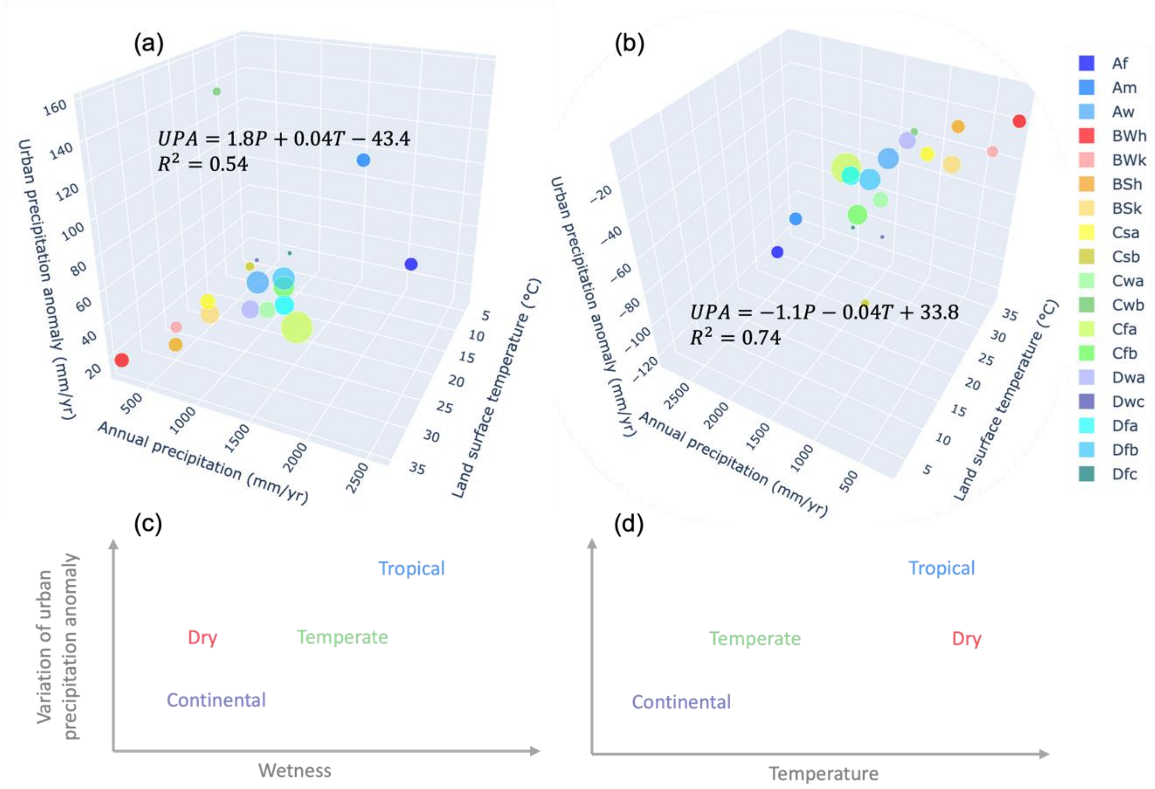

Fig. S2. The relationship between urban precipitation anomalies and local background climates. Three-dimensional scatter plots depicting the average positive (a) and negative (b) urban precipitation anomalies (UPA), background precipitation (P), and land surface temperature (T) for cities in different climate zones. The size of the dots corresponds to the number of cities in each category. Weighted linear regressions and coefficients of determination are provided in the equations. (c and d) Qualitative comparison of the urban precipitation anomalies for cities in various climate zones, (adapted from Fig. 1b). Larger variations in urban precipitation anomalies indicate a larger disparity between urban precipitation and rural precipitation.

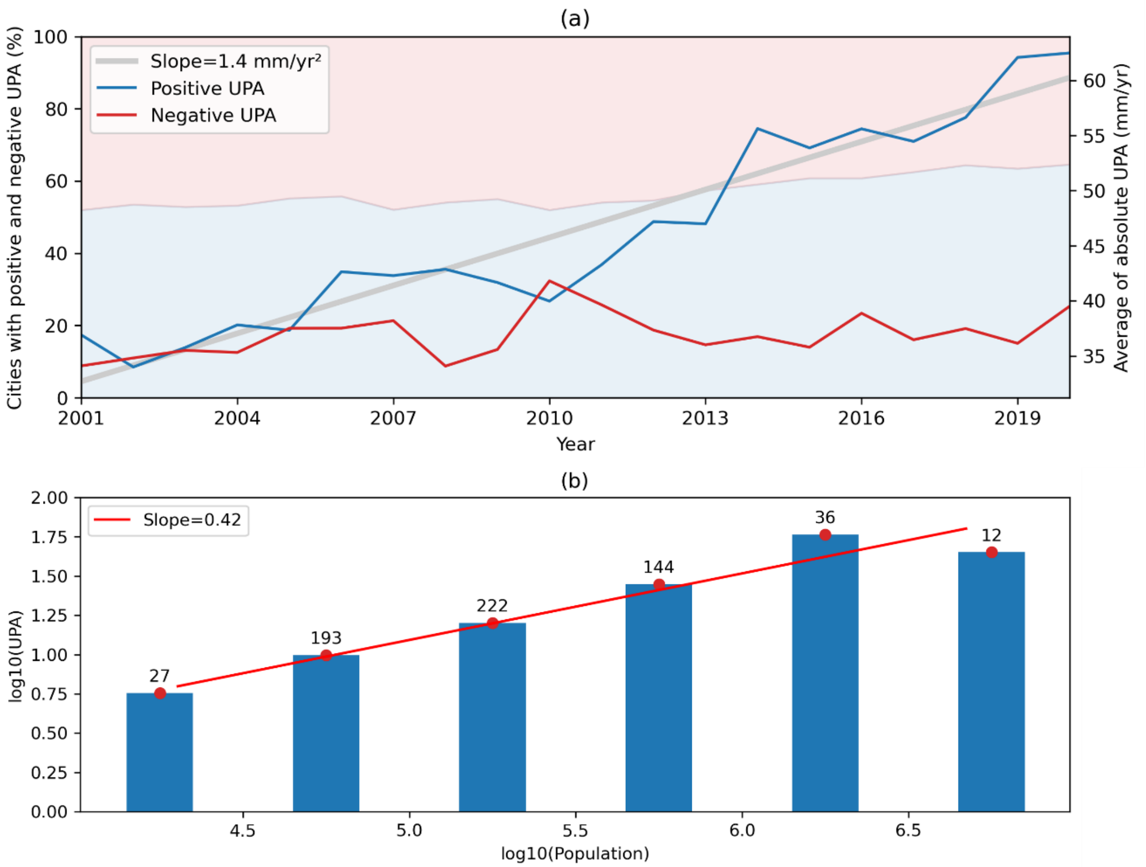

Fig. S3. Exaggerated urban precipitation anomalies with urban development. (a) Changes in urban precipitation anomalies (UPA) from 2001 to 2020. The percentage of cities exhibiting positive UPA increased from 52% to 65% over 20 years. Among these cities, the average positive UPA rose from 37 mm/year to 62 mm/year. (b) Quantitative relationships between UPA and population for cities with positive urban precipitation anomalies. Average urban annual precipitation anomalies in 20 years for cities of varying population groups are presented along the y-axis. The number of cities in each population group is indicated above each bar.

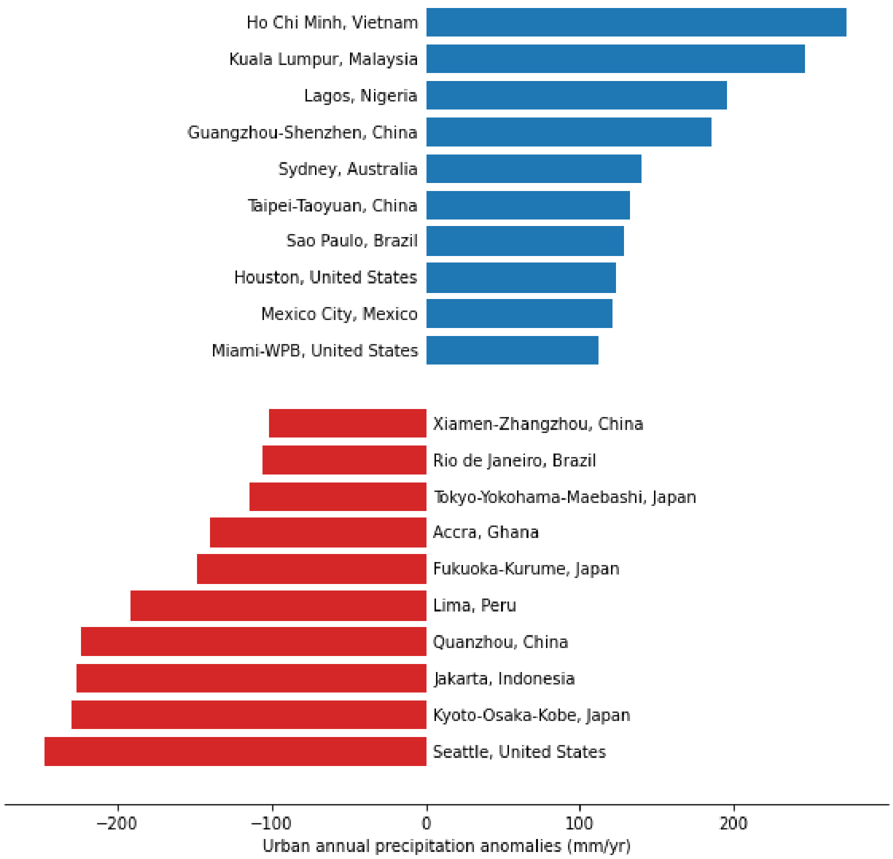

Fig. S4. Top 10 cities with the largest positive and negative urban annual precipitation anomalies among the 100 largest cities in the world.

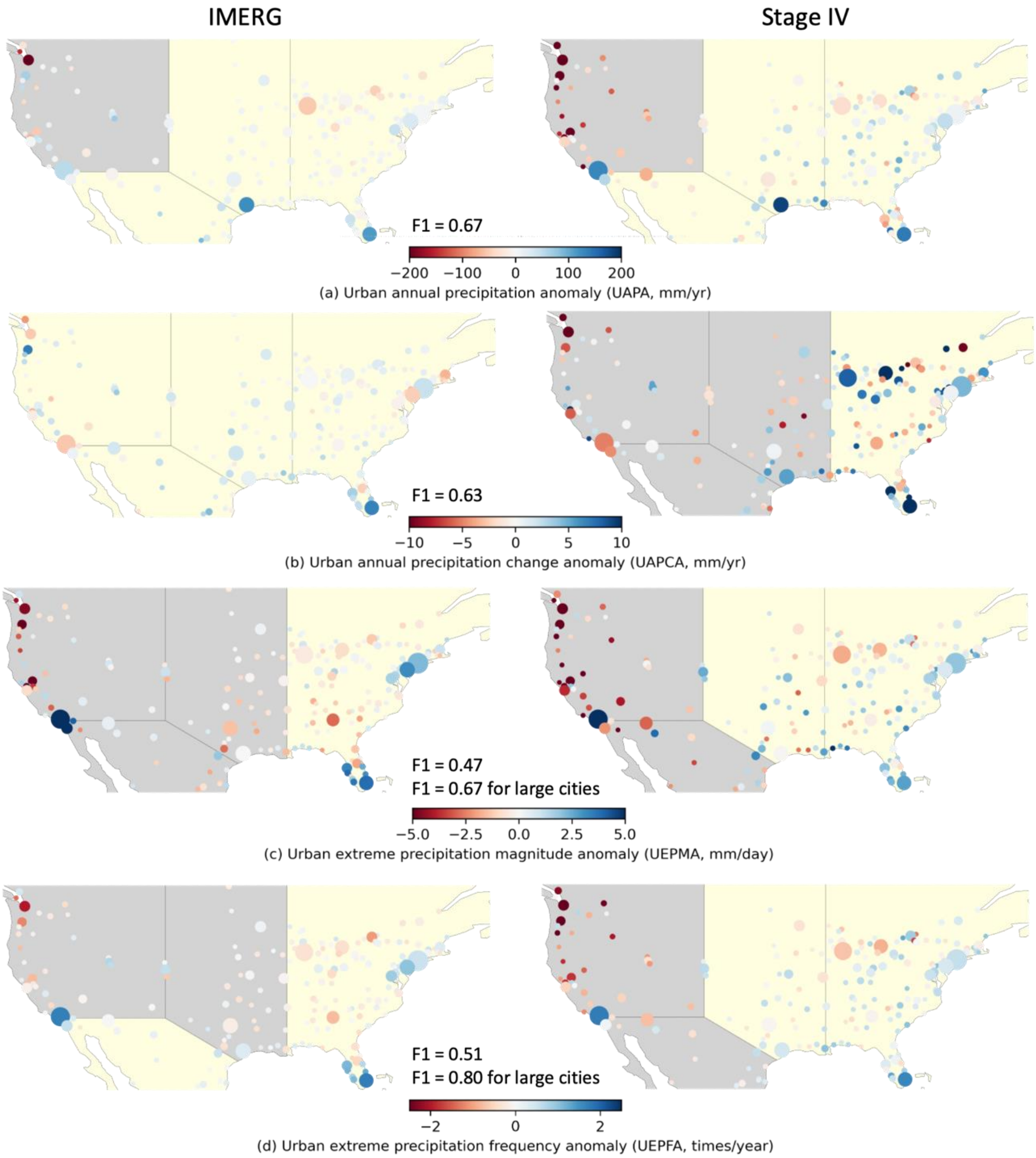

Fig. S5. Comparison of urban annual and extreme precipitation anomalies according to IMERG and Stage IV precipitation datasets for cities in the continental US. IMERG products show better accuracy in estimating annual precipitation anomalies compared to extreme precipitation anomalies. The F1-score for urban annual precipitation and change anomalies exceeds 0.6, while it is approximately 0.5 for both magnitude and frequency anomalies for extreme precipitation. However, IMERG provides more accurate extreme anomalies for 27 large US cities with a city footprint larger than 1000 km^2 with F1-score as 0.67 and 0.8.

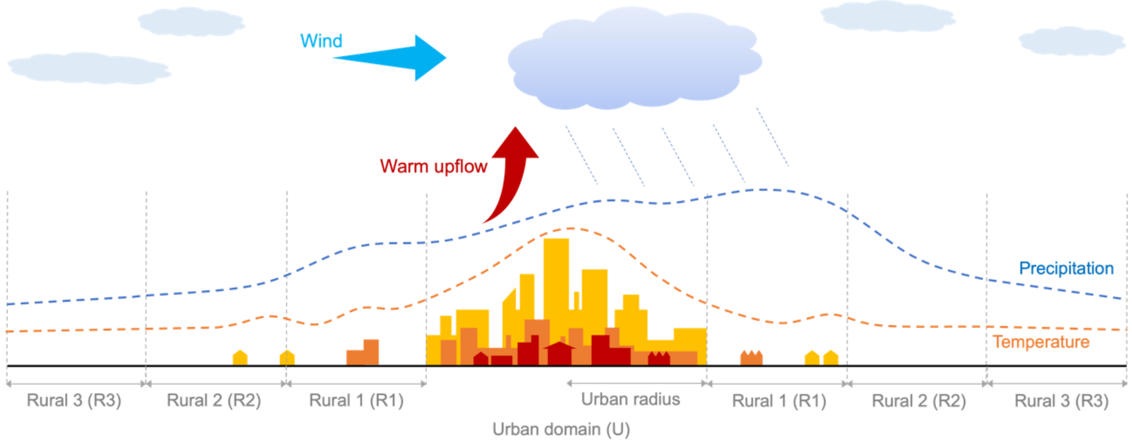

Fig. S6. A schematic illustration of the urban precipitation enhancement over urban and downwind rural areas.

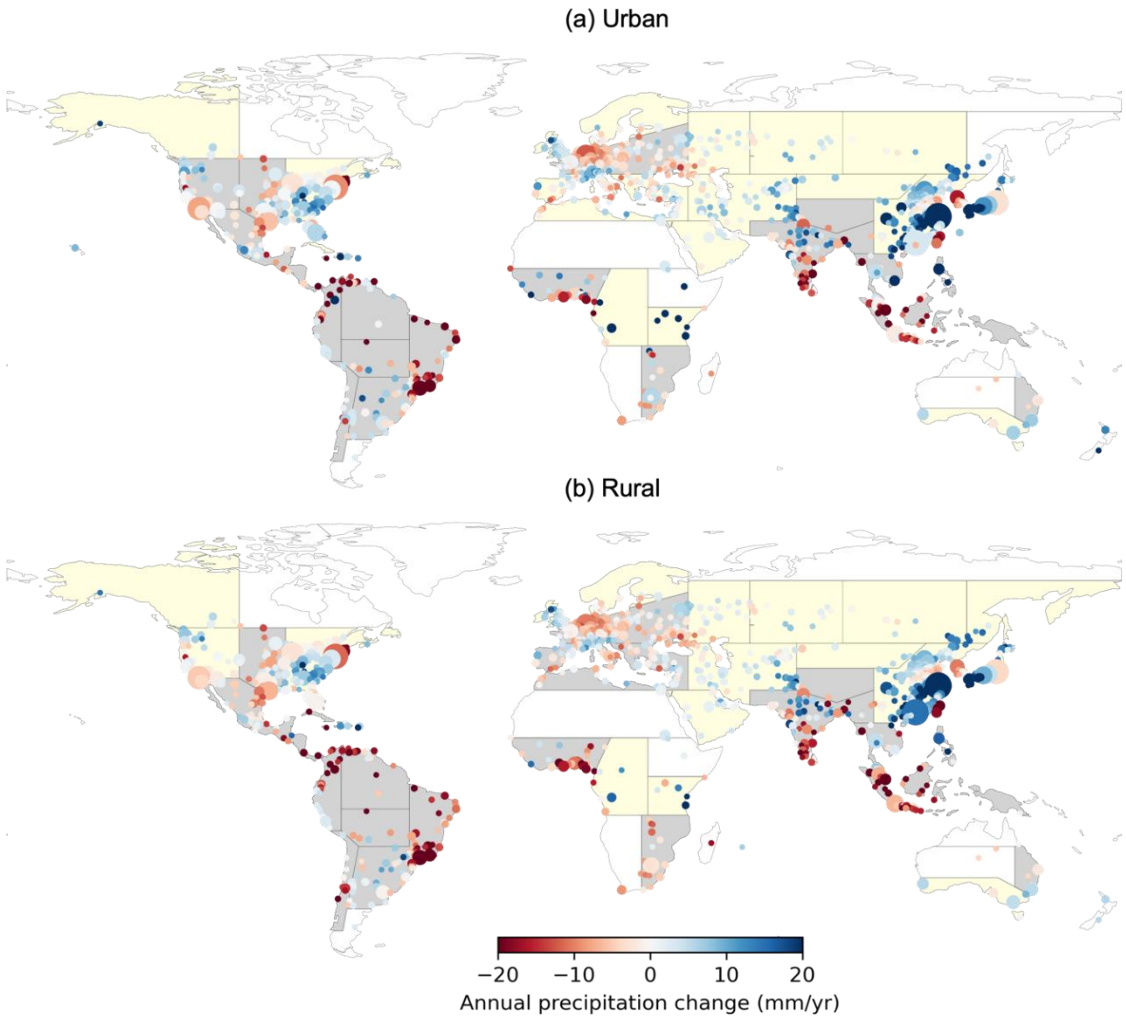

Fig. S7. The annual precipitation changes for (a) global cities and (b) their surrounding rural areas in 20 research years (2001-2020).

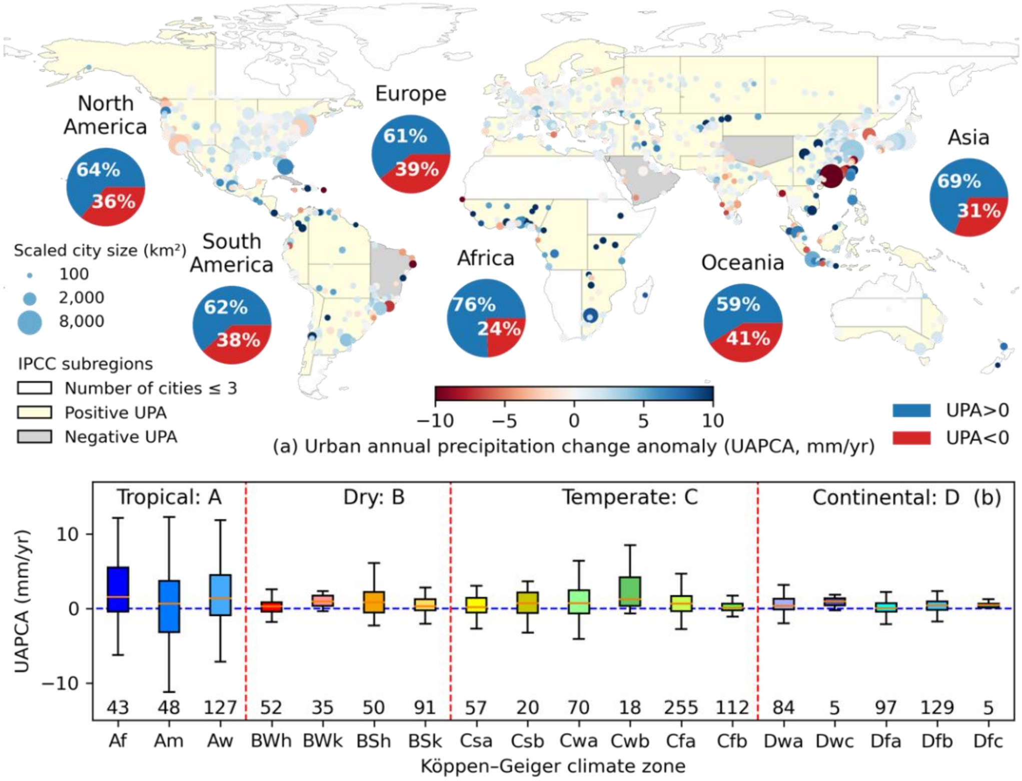

Fig. S8. The urban annual precipitation change anomaly according to (a) continents and (b) climate zones. Similar to Fig. 1. The specific precipitation change in urban and rural areas are shown in Fig S7.

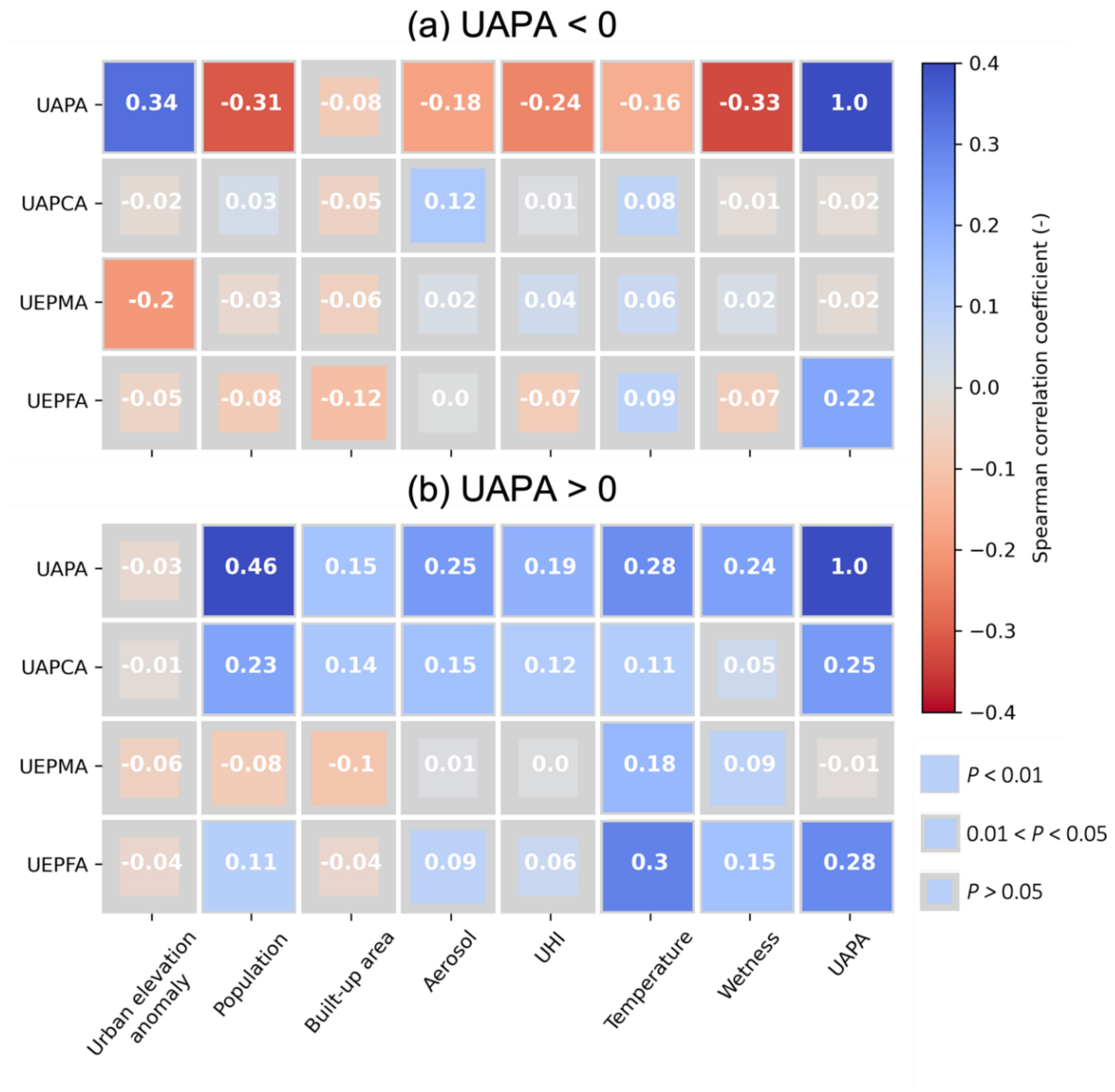

Fig. S9. The Spearman's correlation coefficients between urbanization and environmental factors and urban precipitation anomalies. The cities showing the annual precipitation enhancement (a) and reduction (b) are separated. The color and number on the heat map show Spearman's correlation coefficients between urbanization and environmental factors and urban precipitation anomalies. The significant level is indicated by the P value with the size of colored areas.

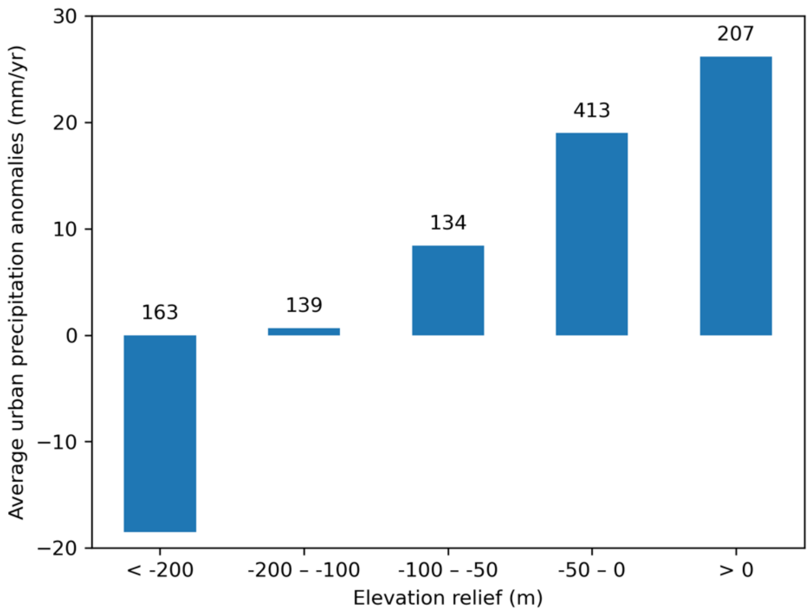

Fig. S10. Urban precipitation anomalies increase with elevation relief. Cities with larger elevation reliefs exhibit larger average urban annual precipitation anomalies. For 163 valley cities with elevation relief smaller than -20 m, the average urban precipitation anomaly is -18.5 mm/year. The number of cities in each population group is indicated above each bar.

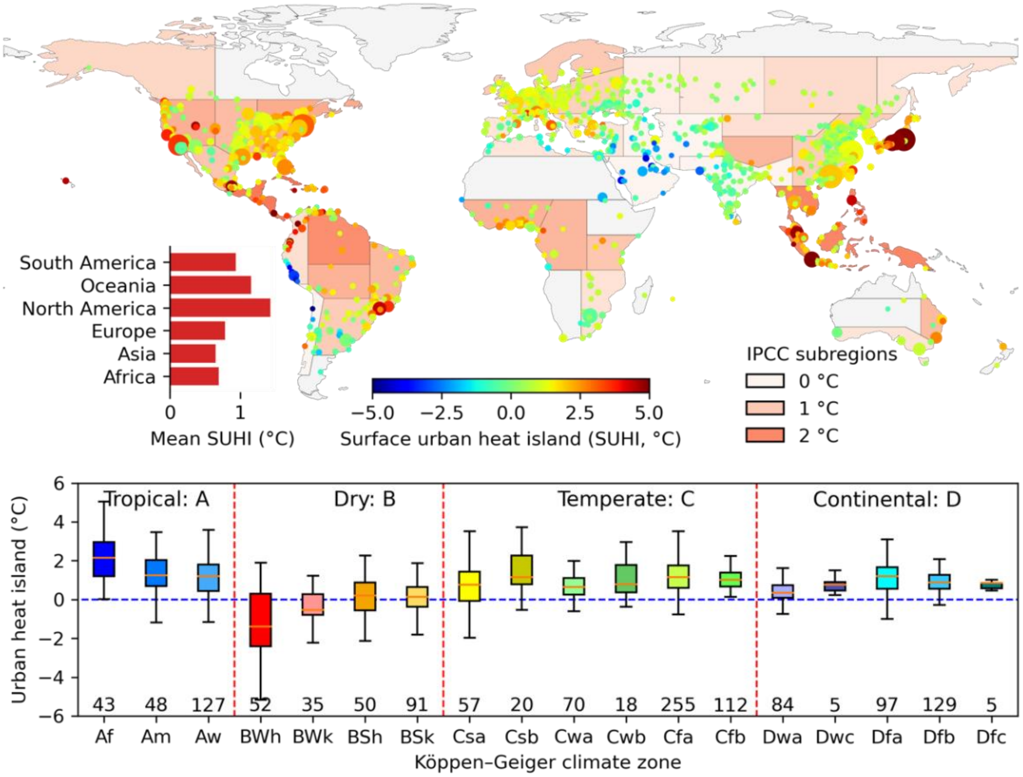

Fig. S11. Similar to Fig. 1, but for the surface urban heat islands (SUHI) according to (a) continents and (b) climate zones. The SUHI demonstrated here is used in the correlation coefficient analysis with urban precipitation anomalies in Fig 4.

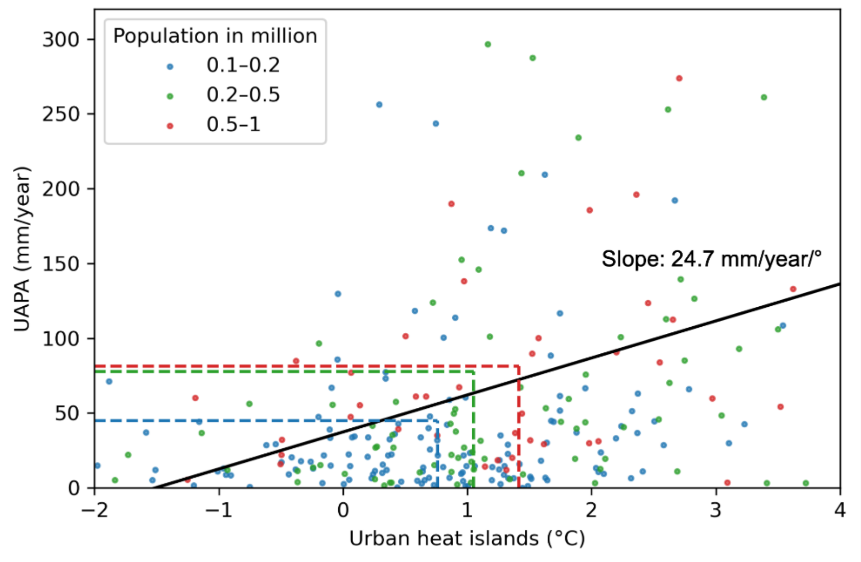

Fig. S12. The scatter plot of urban annual precipitation anomalies and the surface urban heat islands for cities in different population groups in Fig 4a. The solid lines show the linear regression of urban annual precipitation anomalies and the urban heat islands. The dashed lines demonstrate the average values of urban annual precipitation anomalies and urban heat islands for each group.

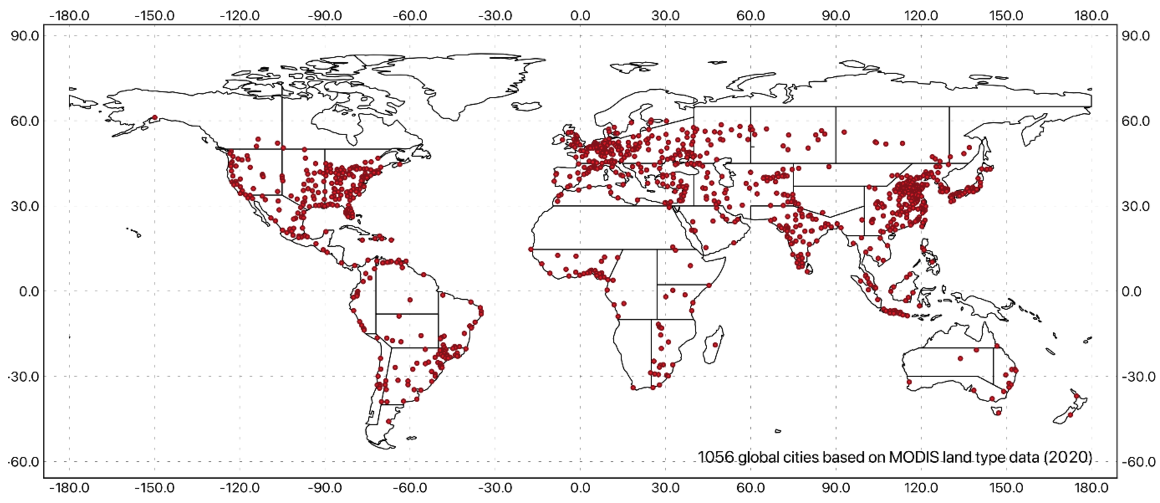

Fig. S13. The location of 1056 global cities in this research

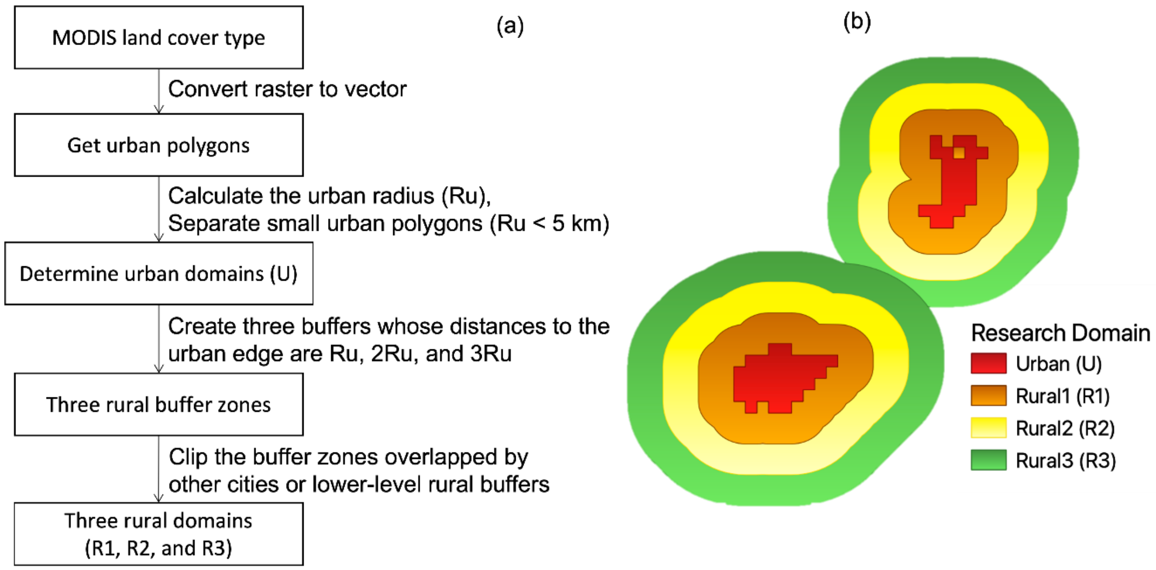

Fig. S14. The method to determine urban and rural domains. (a) The procedure flow chart and (b) the example of research domains for the cities of Austin (upper right) and San Antonio (lower left).

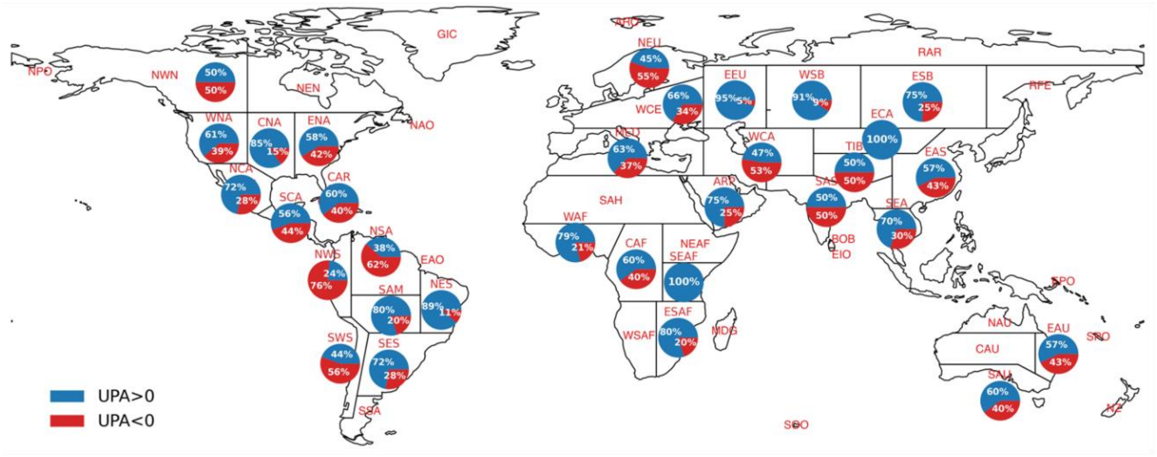

Fig. S15. The percentage of cities with positive and negative urban annual precipitation anomalies according to the Intergovernmental Panel on Climate Change (IPCC) subregions.

Table S1. The seasonal average urban precipitation anomalies for global cities.

	Average UPA in mm/season			Average UPA in percentage		
Season	Positive UPA	Negative UPA	All UPA	Positive UPA	Negative UPA	All UPA
Spring	8.27	-8.22	2.18	4.68	-3.44	1.68
Summer	12.80	-11.03	2.08	6.85	-4.28	1.84
Autumn	11.29	-9.53	4.02	4.44	-3.34	1.72
Winter	9.66	-7.5	2.61	22.92	-5.22	11.33
Whole year	8.94	-7.99	2.72	3.71	-3.02	1.24

Table S2. Overview of two precipitation datasets used in this study.

Product	Full name	Method	Coverage	Temporal resolution	Spatial resolution	Time range	Reference
IMERG	Integrated Multi-satellitE Retrievals for GPM (IMERG) Precipitation V06	Satellite	Globe	Half-hourly	0.1° × 0.1°	2001-nowadays	Huffman et al, 2019[1]
Stage IV	NCEP Stage IV precipitation analysis	Gauge-radar combined	US	Hourly, 6-hourly, 24-hourly	4 km × 4 km	2002-nowadays	Du, 2019[2]

SI References

1. G.J. Huffman, E.F. Stocker, D.T. Bolvin, E.J. Nelkin, J. Tan, GPM IMERG Final Precipitation L3 1 day 0.1 degree x 0.1 degree V06, Edited by Andrey Savtchenko, Greenbelt, MD, Goddard Earth Sciences Data and Information Services Center (GES DISC). Available at 10.5067/GPM/IMERGDF/DAY/06 (2019) Accessed: 08 Sep 2022,
2. J. Du, NCEP/EMC 4KM Gridded Data (GRIB) Stage IV Data. Version 1.0. UCAR/NCAR - Earth Observing Laboratory. Available at https://doi.org/10.5065/D6PG1QDD. (2011) Accessed 08 Sep 2022
